# Deterioration and Manifestation of Cerebral Fat Embolism Triggered by Bone Reaming During Femur Fracture Surgery: A Case Report

**DOI:** 10.1002/ccr3.70806

**Published:** 2025-08-20

**Authors:** Daisuke Nakada, Hideki Hino, Naomi Okuma, Yohei Fujimoto, Masahiro Miyashita, Tadashi Matsuura, Takashi Mori

**Affiliations:** ^1^ Department of Anesthesiology Anesthesia Osaka Metropolitan University Graduate School of Medicine Osaka Japan; ^2^ Osaka Metropolitan University Trauma and Critical Care Center Osaka Japan

**Keywords:** fat embolism syndrome, fracture surgeries, postoperative complication, reaming

## Abstract

The role of intramedullary reaming in triggering fat embolism syndrome (FES) remains debated. This case, where FES developed during reaming, provides further evidence supporting reaming as a potential risk factor for FES.

**Abbreviations**
ARDS: acute respiratory distress syndrome
CFE: cerebral fat embolism
FES: fat embolism syndrome
ICU: intensive care unit
MRI: magnetic resonance imaging
PFO: patent foramen ovale

## 1 Introduction

Fat Embolism Syndrome (FES) represents a severe complication observed in patients with long bone or pelvic fractures. The primary clinical manifestations include central nervous system impairment due to cerebral fat embolism, respiratory distress attributable to acute respiratory distress syndrome (ARDS), and petechial rash. The onset of symptoms typically occurs within 24–72 h post‐injury [1]. To the best of our knowledge, there have been no prior case reports documenting the temporal onset of FES induced by reaming procedures. The potential impact of intramedullary reaming on the development of FES has been a subject of ongoing debate [2]. However, we encountered a case in which FES appeared to develop immediately following intramedullary reaming during surgery. This observation underscores that reaming remains a risk factor for both the occurrence and exacerbation of FES. This report has been prepared in accordance with applicable EQUATOR guidelines, and written informed consent was obtained from the patient prior to publication.

## 2 Case History/Examination

**FIGURE 1:**
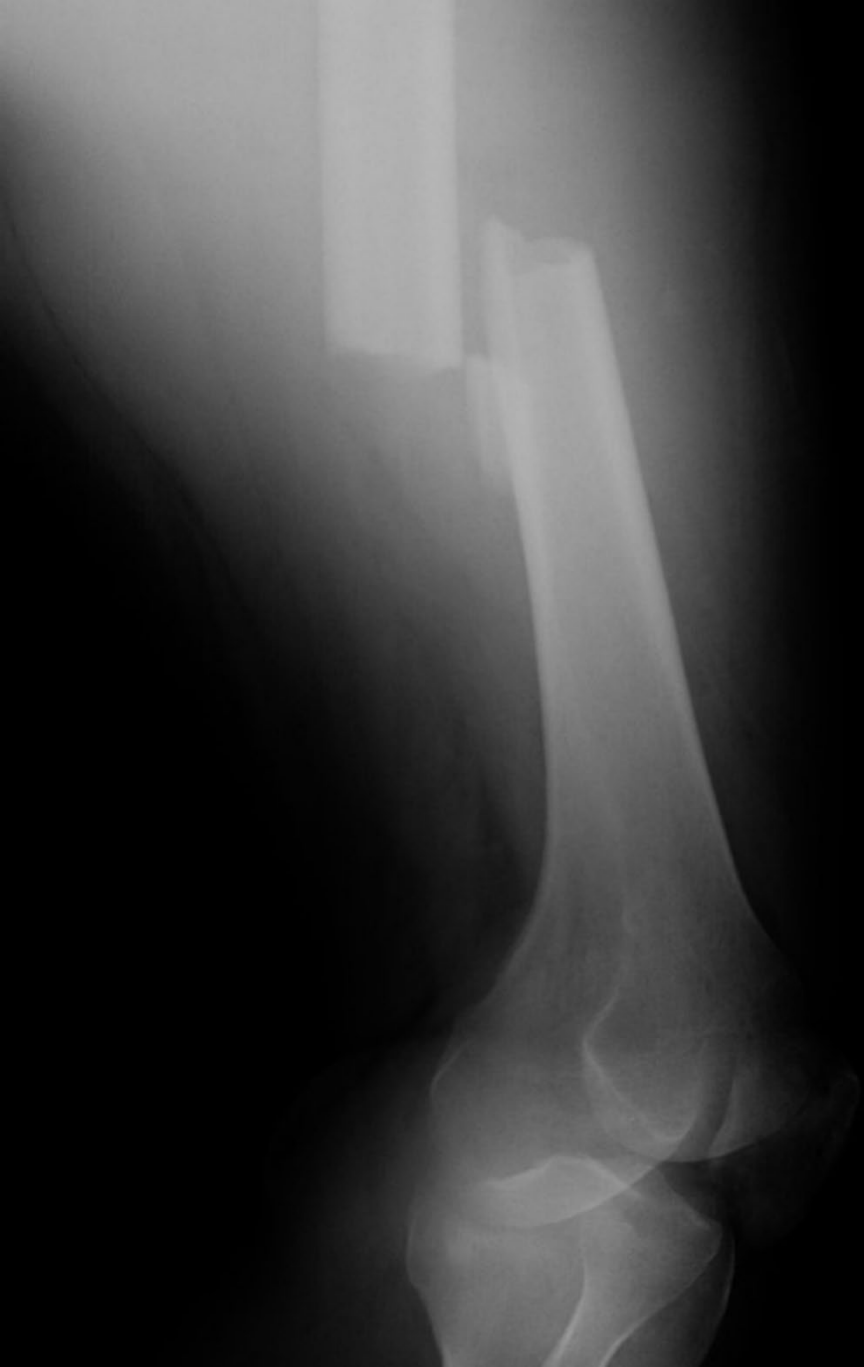
Plain radiograph of the left femur upon admission.

**FIGURE 2:**
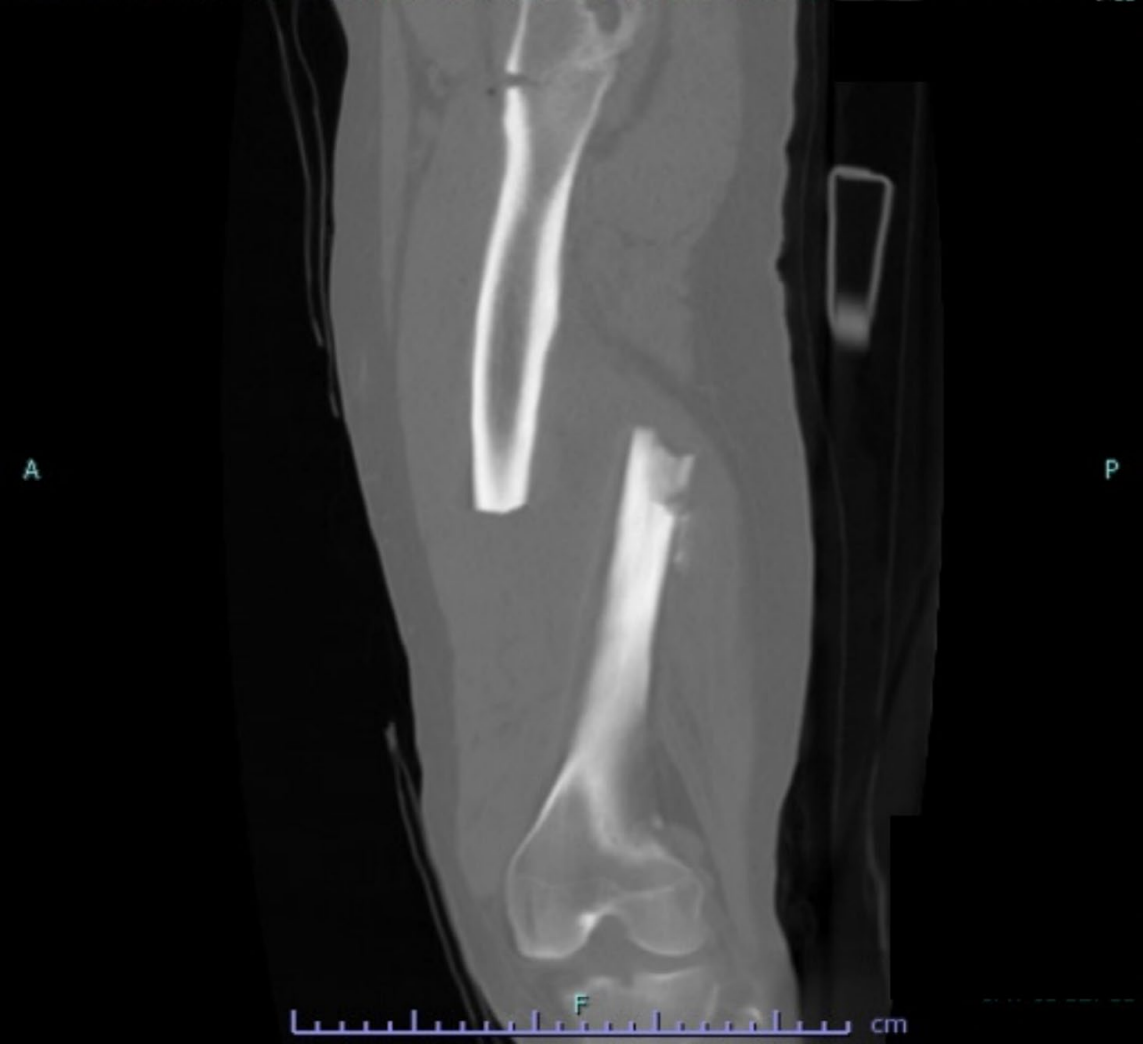
Initial left femur CT scan upon admission.

A previously healthy male in his 40s (height: 176 cm, weight: 100 kg, BMI: 32 kg/m^2^, Medical history: None, Smoking history: None, Alcohol consumption: None, Medication history: None) sustained a left femoral injury following a fall. He experienced high‐energy trauma and was transported to the emergency department. Upon arrival at the center, tachycardia primarily attributed to pain was observed; however, the remaining vital signs were within normal limits. No apparent traumatic injuries to the head or chest were identified. The left lower extremity exhibited significant swelling, pain, and external rotation without any open wounds. Laboratory findings revealed leukocytosis (10,800/μL) and elevated fibrin degradation products (26.3 mg/mL). Leukocytosis may indicate inflammation or stress from trauma, while elevated fibrin degradation products could suggest systemic activation of coagulation. Arterial blood gas analysis results were within normal ranges (pH: 7.399, PaO_2_: 77.4 mmHg, PaCO_2_: 42.4 mmHg). Plain X‐ray and computed tomography (CT) revealed fractures of the left femoral shaft and trochanteric region (Figures 1 and 2). No other obvious traumatic changes or thrombotic events were detected in other areas.

## 3 Methods

Following admission to the intensive care unit (ICU), the fractures were managed with traction, and surgery was scheduled for the third day. The patient had sustained ipsilateral fractures of the femoral trochanteric region and femoral shaft, and intramedullary nailing was planned. The patient remained alert until the second day; however, disorientation emerged on the morning of the third day. Due to inadequate pain control, this altered mental status was initially attributed to delirium, and the patient was transferred to the operating room for the scheduled fracture repair. At that time, although disoriented, the patient was able to follow simple commands.

**FIGURE 3:**
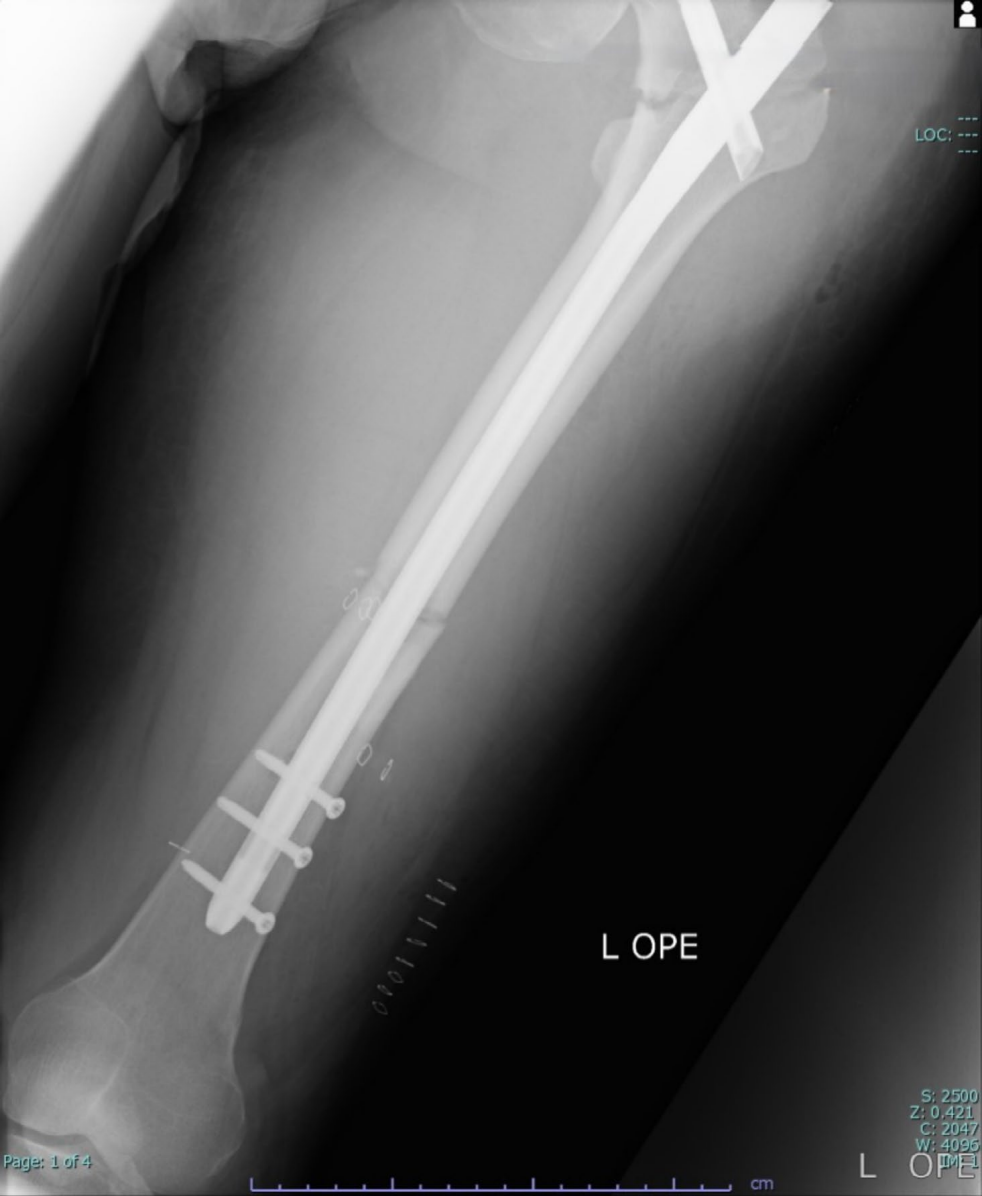
Postoperative plain radiograph demonstrating fixation of the left femoral shaft and trochanteric fractures.

During surgery, continuous phenylephrine infusion was required to manage persistent hypotension accompanied by tachycardia. Notably, the dose requirement increased immediately after reaming, which was not attributable to hemorrhage but rather indicative of reduced systemic vascular resistance caused by FES. While SpO_2_ was maintained at 100% with 50% oxygen, it transiently decreased to approximately 96%–97% at the initiation of reaming. The procedure lasted 2 h and 59 min, with an estimated blood loss of 260 mL. The prolonged operative time was due to the challenges associated with the reduction and fixation of both the ipsilateral trochanteric and diaphyseal fractures using an intramedullary nail, which was further complicated by the patient's obesity. Postoperative radiographic imaging demonstrated successful fixation of the fractures (Figure 3).

Upon emergence from general anesthesia, the patient was extubated. However, the patient exhibited delirium and agitation, making it difficult to follow commands. MRI confirmation was challenging due to the patient's uncooperative movements. The patient was subsequently admitted to the ICU.

**FIGURE 4:**
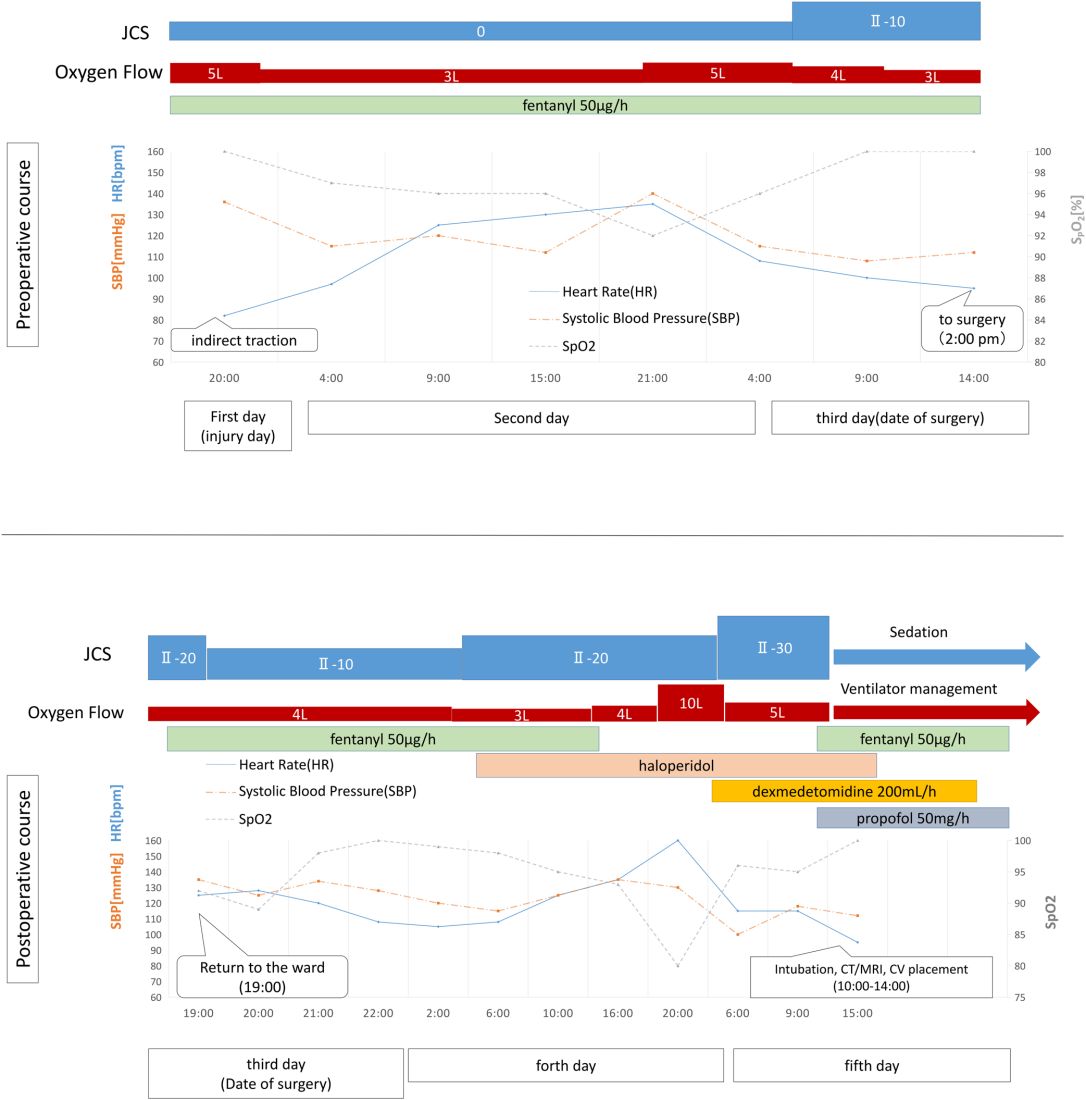
Perioperative records.

**FIGURE 5:**
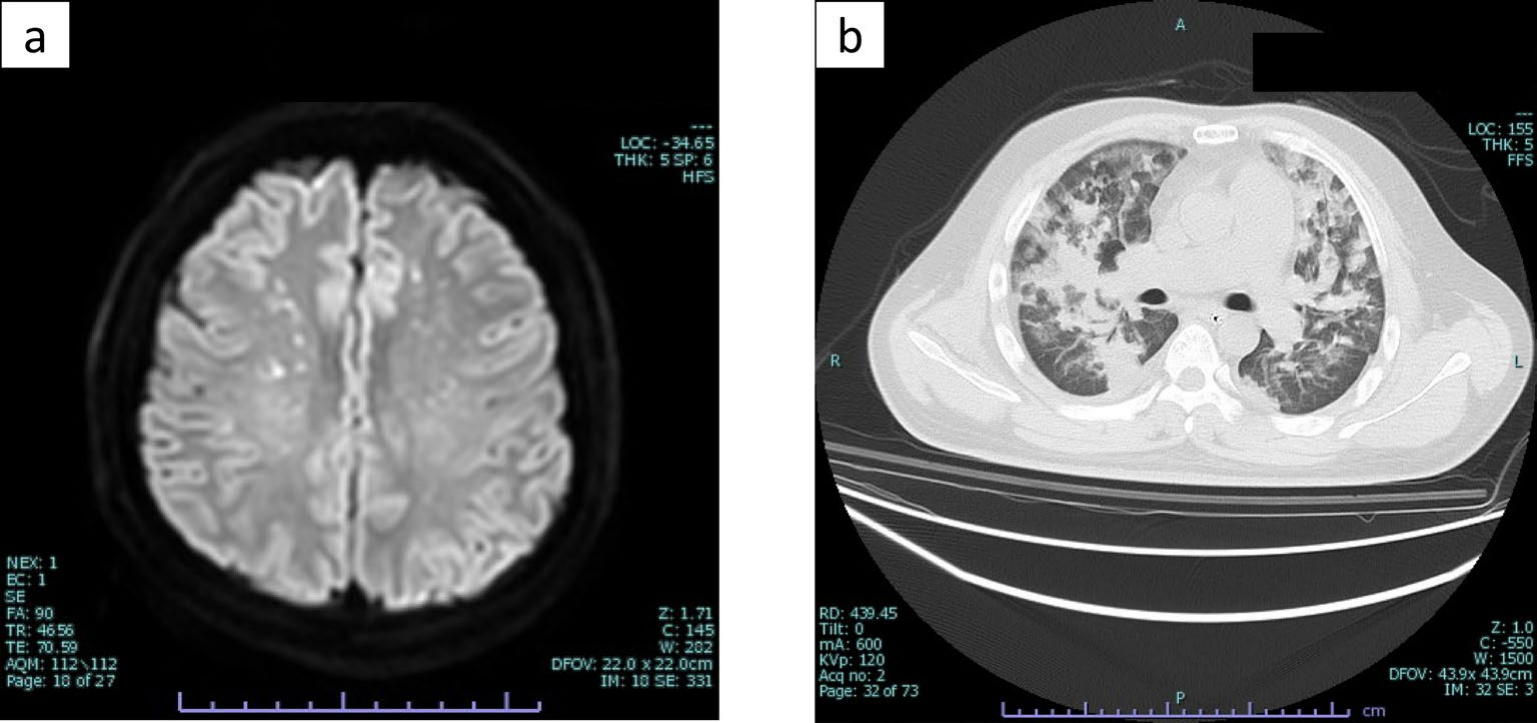
Perioperative images during the course of treatment. (a) Head MRI on Day 5 shows “starfield pattern”; (b) Chest CT on Day 5 shows signs of acute respiratory distress syndrome. CT, computed tomography; MRI, magnetic resonance imaging.

**FIGURE 6:**
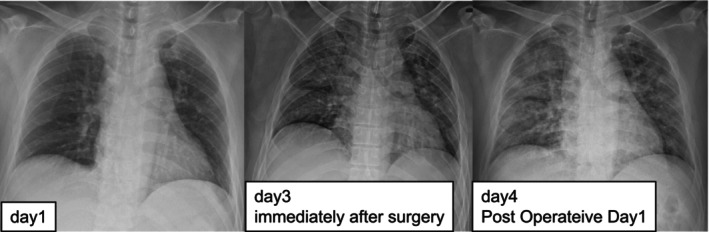
Chest X‐ray image over time.

Postoperatively, haloperidol was administered to manage agitation. On the first postoperative day, the patient developed respiratory distress, became semicomatose, and exhibited progressive clinical deterioration (Figure 4). Endotracheal intubation and mechanical ventilation were initiated. Diffusion‐weighted MRI revealed multiple high‐intensity lesions in a characteristic “starfield” pattern, consistent with cerebral fat embolism (CFE). Additionally, chest radiography and CT imaging showed bilateral pulmonary infiltrates suggestive of ARDS (Figures 5 and 6).

Differential diagnoses considered included neurogenic pulmonary edema, diffuse alveolar hemorrhage, aspiration pneumonia, and cardiogenic pulmonary edema. The characteristic MRI findings, together with Gurd and Wilson's criteria, supported a diagnosis of FES with CFE involvement. Neurogenic pulmonary edema was considered less likely given its typically acute onset and characteristic central distribution pattern on imaging. Diffuse alveolar hemorrhage was also deemed unlikely due to the absence of clinical indicators such as hemoptysis and progressive anemia. Aspiration pneumonia was considered, but the diffuse, homogeneous distribution of pulmonary infiltrates observed was more consistent with ARDS. Cardiogenic pulmonary edema was judged unlikely based on the patient's hemodynamic presentation of low vascular resistance shock.

## 4 Conclusion and Results

Methylprednisolone (1 mg/kg/day) was initiated as treatment for ARDS associated with FES, resulting in gradual symptom improvement. The methylprednisolone regimen was continued for 10 days. The administration of methylprednisolone was considered to have prevented the second‐hit phenomenon of FES and the exacerbation of ARDS. By Day 7, the patient's level of consciousness gradually improved, and communication became possible. The patient was extubated on Day 9 and began rehabilitation the following day, with an initial goal of non‐weight‐bearing ambulation using crutches. The patient was discharged from the ICU to the ward on Day 10. By Day 16, the patient was able to ambulate with crutches, although weight‐bearing remained restricted. However, residual higher‐order cognitive dysfunction posed challenges during rehabilitation. On Day 17, the patient was transferred to a rehabilitation hospital for further recovery. After hospital discharge, outpatient follow‐up continued until the patient underwent implant removal surgery two and a half years postoperatively. Subsequently, the patient successfully returned to society without functional impairments.

## 5 Discussion

FES is relatively rare but a serious complication of fractures, and the chance of it developing has been reported as 0.8%–23% [1]. Risk factors for FES include young age, particularly males aged 10–39 years, multiple fractures, closed fractures, delayed treatment of diaphyseal fractures, dehydration, impaired cardiopulmonary function, and pulmonary injuries [3, 4, 5, 6]. The presence of a patent foramen ovale (PFO), a known risk factor for paradoxical cerebral embolism, is not a prerequisite for the development of FES. Fat droplets have been reported to pass through pulmonary capillaries and reach cerebral vessels [7], and cases of FES without PFO have been documented. In this case, identified risk factors included male sex, multiple fractures, closed fractures, diaphyseal fractures, and delayed treatment, with FES manifesting intraoperatively.

Reaming, a potential additional factor contributing to FES, played a role in this case. Intraoperative diaphyseal reaming has been reported as a risk factor for FES, as it can dislodge fat droplets from the bone marrow into the bloodstream, increase pulmonary artery pressure, and decrease PaO_2_ [8]. Although the extent to which reaming influences FES development remains debated [9], it has been suggested that the excessive inflammatory response stimulated by intramedullary reaming may trigger FES [2].

The decision to use reaming in intramedullary nailing procedures remains controversial. In patients with high infection risk, such as those with open fractures, non‐reamed techniques are often preferred to reduce surgical site infections. However, non‐reamed techniques are believed to provide inferior fixation stability [10]. In this case, alternative fixation methods would have required the use of a short intramedullary nail for the trochanteric region and plate fixation for the diaphyseal fracture. However, this approach would have resulted in significantly longer operative time and greater surgical invasiveness, making it impractical. Therefore, intramedullary nailing was chosen as the preferred fixation method. Considering the increased risk of nonunion associated with non‐reamed techniques, a gentle reaming approach was employed, performing the minimal necessary reaming to balance stability and procedural safety.

Timing of surgery is another debated topic. Pape et al. [11] reported that patients with femoral fractures and concurrent thoracic trauma who underwent internal fixation within 24 h had significantly higher rates of ARDS and mortality compared to those treated after 24 h. Conversely, Enninghorst et al. found that early internal fixation was safe and reduced complications in patients without multiple trauma [12]. Based on these findings, the EAST (Eastern Association for the Surgery of Trauma) guidelines conditionally recommend internal fixation within 24 h [13]. In this case, the absence of trauma outside the fracture sites and suspected delirium due to poor pain control suggested that early internal fixation might have been a preferable strategy to mitigate the risk of FES.

Diagnosing FES is challenging due to its typically nonspecific symptoms. Several diagnostic criteria exist, yet no single set is considered superior. Zenker first described FES in 1862, and Gurd proposed diagnostic criteria in 1970 [14, 15], which were later revised by Gurd and Wilson in 1974 [16]. While various other criteria have been proposed, they suffer from being highly nonspecific because most symptoms also occur in other clinical trauma settings. Furthermore, they have not been prospectively validated in sufficiently large cohorts, making their routine use in clinical practice not ideal [5]. The “starfield pattern” observed on brain MRI is a characteristic finding of FES [17]. Given the nonspecific nature of FES, clinicians should maintain a high index of suspicion and actively pursue diagnostic evaluation. In cases where FES is suspected, brain MRI is valuable for confirmation. Although multiple cerebral infarctions and postoperative ARDS were considered in this case, the characteristic MRI pattern supported the diagnosis of FES with associated CFE.

In conclusion, the hemodynamic changes observed immediately after reaming suggest that reaming is a risk factor for FES. However, as this report represents a single case, further accumulation of cases is necessary to strengthen this observation.

## Author Contributions

**Daisuke Nakada:** writing – original draft (lead); investigation (supporting). **Hideki Hino:** writing – review and editing (equal). **Naomi Okuma:** investigation (lead). **Yohei Fujimoto:** investigation (supporting). **Masahiro Miyashita:** investigation (lead). **Tadashi Matsuura:** writing – review and editing (equal). **Takashi Mori:** writing – review and editing (equal). All authors read and approved the final manuscript.

## Ethics Statement

The authors have nothing to report.

## Consent

Written consent was obtained from the patient for publication of this case report and accompanying images.

## Conflicts of Interest

The authors declare no conflicts of interest.

## Data Availability

The data that support the findings of this study are openly available in Figshare at [10.6084/m9.figshare.29210816].
